# Peptide mimic for influenza vaccination using nonnatural combinatorial chemistry

**DOI:** 10.1172/JCI91512

**Published:** 2018-03-12

**Authors:** John J. Miles, Mai Ping Tan, Garry Dolton, Emily S.J. Edwards, Sarah A.E. Galloway, Bruno Laugel, Mathew Clement, Julia Makinde, Kristin Ladell, Katherine K. Matthews, Thomas S. Watkins, Katie Tungatt, Yide Wong, Han Siean Lee, Richard J. Clark, Johanne M. Pentier, Meriem Attaf, Anya Lissina, Ann Ager, Awen Gallimore, Pierre J. Rizkallah, Stephanie Gras, Jamie Rossjohn, Scott R. Burrows, David K. Cole, David A. Price, Andrew K. Sewell

**Affiliations:** 1 Centre for Biodiscovery and Molecular Development of Therapeutics, Australian Institute of Tropical Health and Medicine, James Cook University, Cairns, Queensland, Australia.; 2 QIMR Berghofer Medical Research Institute, Brisbane, Queensland, Australia.; 3 Division of Infection and Immunity, Cardiff University School of Medicine, Cardiff, United Kingdom.; 4 School of Medicine, The University of Queensland, Brisbane, Queensland, Australia.; 5 Griffith University, Brisbane, Queensland, Australia.; 6 Systems Immunity Research Institute, Cardiff University, Cardiff, United Kingdom.; 7 School of Biomedical Sciences, The University of Queensland, Brisbane, Queensland, Australia.; 8 Infection and Immunity Program and Department of Biochemistry and Molecular Biology, Biomedicine Discovery Institute, and; 9 Australian Research Council Centre of Excellence in Advanced Molecular Imaging, Monash University, Clayton, Victoria, Australia.; 10 Human Immunology Section, Vaccine Research Center, National Institute of Allergy and Infectious Diseases, NIH, Bethesda, Maryland, USA.

**Keywords:** Immunology, Infectious disease, Influenza, Peptides, T cells

## Abstract

Polypeptide vaccines effectively activate human T cells but suffer from poor biological stability, which confines both transport logistics and in vivo therapeutic activity. Synthetic biology has the potential to address these limitations through the generation of highly stable antigenic “mimics” using subunits that do not exist in the natural world. We developed a platform based on D–amino acid combinatorial chemistry and used this platform to reverse engineer a fully artificial CD8^+^ T cell agonist that mirrored the immunogenicity profile of a native epitope blueprint from influenza virus. This nonnatural peptide was highly stable in human serum and gastric acid, reflecting an intrinsic resistance to physical and enzymatic degradation. In vitro, the synthetic agonist stimulated and expanded an archetypal repertoire of polyfunctional human influenza virus–specific CD8^+^ T cells. In vivo, specific responses were elicited in naive humanized mice by subcutaneous vaccination, conferring protection from subsequent lethal influenza challenge. Moreover, the synthetic agonist was immunogenic after oral administration. This proof-of-concept study highlights the power of synthetic biology to expand the horizons of vaccine design and therapeutic delivery.

## Introduction

CD8^+^ T cells recognize short peptide fragments presented by MHC class I (MHC-I) molecules on the surface of nucleated cells (1–3). These peptide–MHC-I (pMHC-I) molecular arrays are scanned by clonotypically distributed αβ T cell receptors (TCRs) (4), which trigger T cell activation beyond a preset monomeric TCR/pMHC-I affinity threshold (5–8). This process enables the immune system to identify and eliminate infected and abnormal cells via targeted cytotoxicity, while remaining inert in the presence of healthy cells expressing a repertoire of unaltered self-derived peptides. Attenuated whole organisms, protein subunits, and/or peptides are typically used in vaccine formulations to prime immune responses against various cancers and dangerous pathogens. In the setting of infectious disease alone, prophylactic vaccines are thought to prevent approximately 9 million deaths annually (9). However, effective prophylaxis is lacking for most human diseases, and the global economic burden of current operational vaccines is high, costing around $4 billion annually (10). In particular, the temperature-controlled supply chain for these sensitive biological compounds can account for up to 80% of the total deployment cost (11). Environmental stability is therefore a strategic priority for current vaccine research and development.

Synthetic biology can be described as the design and refabrication of existing biological systems using nonnatural components. The vast majority of proteins in nature are constructed from L–amino acids, which are highly susceptible to degradation by endogenous and environmental proteases. In contrast, D–amino acids are rarely found in nature and typically exist as point mutations in L-polypeptide chains, for example in prokaryotic cell walls, bacterial antibiotics, certain animal proteins and venoms, and neuroregulators in the human brain (12–16). Although D–amino acids are mirror image stereoisomers of L–amino acids with identical chemical and physical properties, the corresponding proteins are intrinsically resistant to protease-mediated hydrolysis (13). Immunogens designed from these building blocks may therefore allow the production of stable vaccines with enhanced bioavailability and in vivo efficacy. Additional benefits include the potential for therapeutic activity via oral ingestion.

Large-scale T cell scanning studies using combinatorial peptide libraries (CPLs) (17–19) and yeast-displayed pMHC libraries (20) have shown that cross-reactivity is an inherent property of TCRs (reviewed in ref. 21). Accordingly, it may be feasible to generate nonnatural D–amino acid agonists that mimic their native counterparts (22, 23). In this study, we synthesized a nonamer CPL using only D–amino acid subunits to reverse engineer a fully synthetic agonist in the setting of a relevant human disease. The data validate what we believe to be a novel and systematic approach to the design of nonnatural immunogens that offers substantial advantages over current vaccine formulations.

## Results

### Identification of D–amino acid agonists via CPL screening.

Our first task was to design a system that allowed robust and reproducible testing of synthetic T cell agonists in vitro and in vivo using a disease-relevant target. Influenza A virus was selected as an expedient model for this purpose, because antigen-specific memory T cell populations are commonly present in adult humans, and pathogen challenge experiments are feasible in humanized mice. The blueprint for synthetic agonist design was the immunodominant HLA-A*0201–restricted GILGFVFTL_58–66_ (GIL) peptide derived from the influenza matrix protein (M1). Based on previous reports of occasional cross-recognition (reviewed in ref. 22), we initially examined the ability of a retroinversion of the GILGFVFTL epitope ltfvfglig (lower case type used to denote D–amino acids) to activate an archetypal TRBV19^+^ (24) HLA-A2–GILGFVFTL–specific CD8^+^ T cell clone (ALF3). In this particular setting, however, the retroinverted D peptide was not immunogenic (Supplemental Figure 1A; supplemental material available online with this article; https://doi.org/10.1172/JCI91512DS1). These observations were not entirely surprising, given the paucity of examples of immunogenic D–amino acid retroinversion T cell agonists described to date (22).

**Figure 1:**
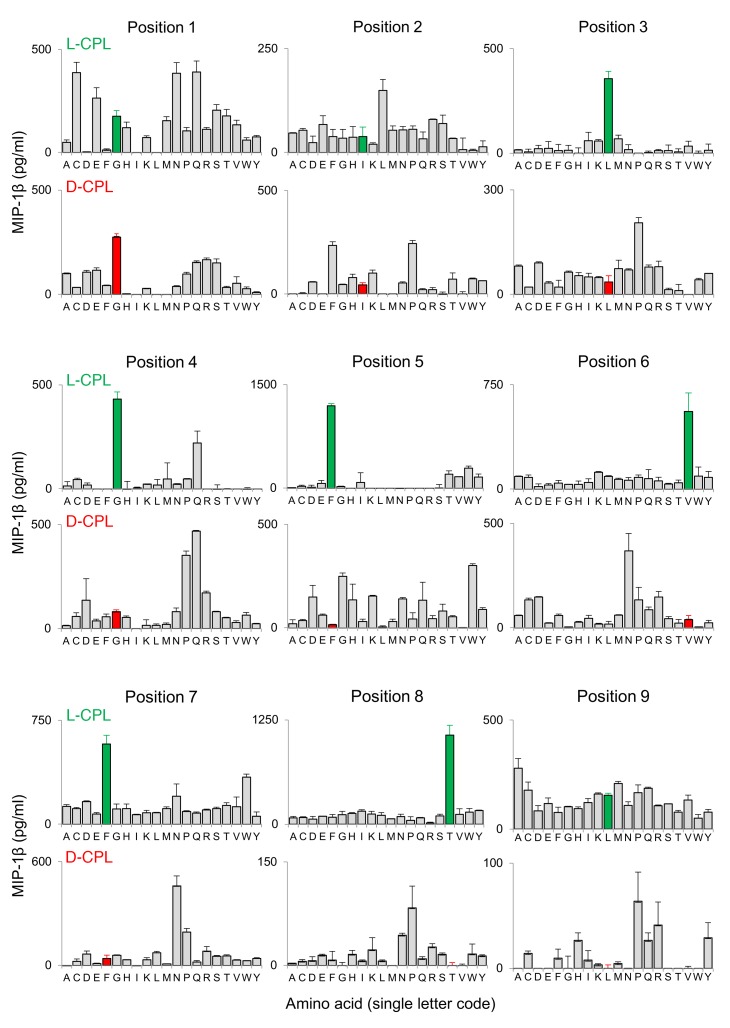
An archetypal human CD8^+^ T cell clone exhibits broad but differing L– and D–amino acid recognition profiles. Clonal ALF3 CD8^+^ T cells were incubated with C1R-A2 target cells pulsed with CPL mixtures (100 μM) comprising nonamer L– or D–amino acids. MIP-1β release in the supernatants was quantified by ELISA. The amino acid residue in each position corresponding to the index GILGFVFTL peptide is depicted in green for the L-CPL screen and red for the D-CPL screen. Fixed amino acid positions (single letter code) along the peptide backbone are indicated. Error bars from 2 replicates depict SEM.

In subsequent experiments, we used CPL scanning to conduct a systematic search for nonnatural D–amino acid agonists capable of triggering HLA-A2–GILGFVFTL–specific CD8^+^ T cells. This approach has been employed successfully in the past to identify and augment L–amino acid ligands (18, 19, 25, 26). A novel D–amino acid nonamer CPL was synthesized and used in positional scanning format to screen the ALF3 clone (Figure 1), selected to represent a common bias toward TRBV19 gene usage within GILGFVFTL-specific memory CD8^+^ T cell populations (24, 27, 28). Of note, the D–amino acid CPL was length-matched to the GILGFVFTL peptide, given previous data showing that MHC-I–restricted TCRs are preprogrammed to engage bound ligands spanning a defined number of residues (19). Surprisingly, the L– and D–amino acid scans revealed very different recognition patterns across the 180 peptide mixtures, indicating that D–amino acid agonists cannot be predicted from their known biological counterparts. These parallel scans also suggested that the ALF3 clone recognized at least as many D–amino acid agonists as L–amino acid agonists, further highlighting the vast cross-reactive potential of αβ TCR surveillance. Moreover, the L–amino acid scan data were similar to those generated with other TRBV19^+^ GILGFVFTL-specific CD8 T cell clones, indicating that T cells with different TCRs with similar antigen specificities generated related cross-reactivity profiles (data not shown).

**Figure 2:**
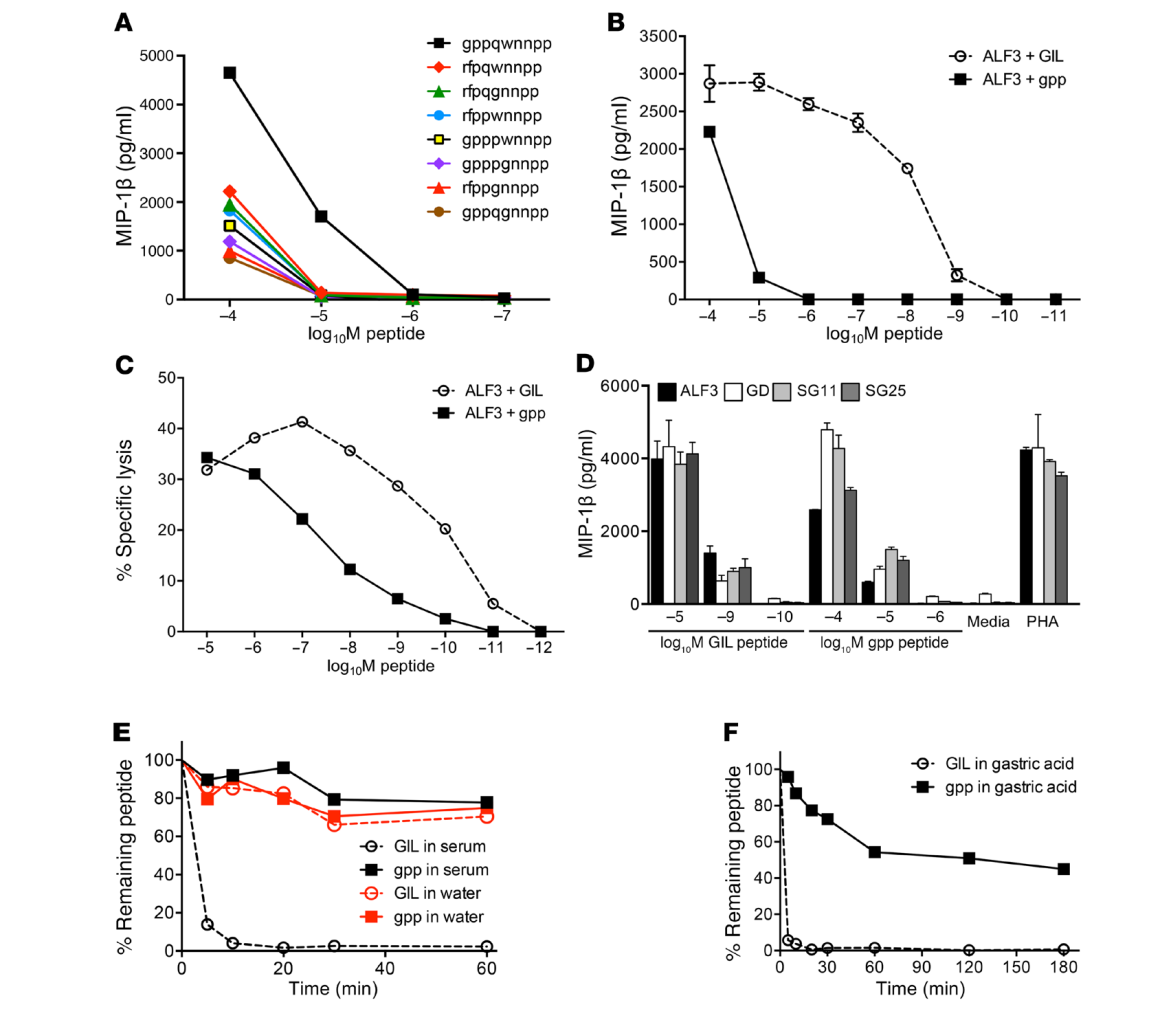
A fully synthetic agonist designed from CPL scan data is recognized by multiple influenza-specific clones and is highly resistant to human proteases and gastric acid. (**A**) Clone ALF3 was incubated with C1R-A2 target cells pulsed with the indicated concentrations of D–amino acid candidate agonists predicted from the D–amino acid CPL scan (Figure 1). MIP-1β release in the supernatants was quantified by ELISA. Errors from 2 replicates depict SEM. (**B**) ALF3 was incubated overnight with the indicated concentrations of GILGFVFTL and gppqwnnpp. Errors from 2 replicates depict SEM. (**C**) Chromium release cytotoxicity assay using ALF3 and CIR-A2 targets incubated with gppqwnnpp and GILGFVFTL peptides at the concentrations shown. Errors from 2 replicates depict SEM. (**D**) As in **A** but including GILGFVFTL with clones GD, SG11, and SG25. ALF3 was also included for comparison. Errors from 2 replicates depict SEM. (**E**) The GILGFVFTL or gppqwnnpp peptides were added to human serum or MilliQ water and sampled in triplicate at the indicated time points. Ion peak signals that identified each peptide were quantified using LCMS. Stability was calculated as the area percentage of each serum-treated or water-treated ion peak relative to the same ion peak at 0 minutes. (**F**) GILGFVFTL and gppqwnnpp were added to simulated gastric acid (NaCl, pepsin, and HCl; pH 1.2) and sampled in triplicate at the indicated time points. Ion peak signals that identified each agonist were quantified using LCMS. Stability was calculated as the area percentage of each gastric acid–treated ion peak relative to the same ion peak at 0 minutes. Recovery rates of gppqwnnpp in human serum and gastric acid were significantly higher compared with GILGFVFTL at all time points beyond 0 minutes (*P* < 0.00001). Errors from 3 replicates depict SEM. In some panels, error bars are smaller than the plot symbols.

Informed by these quantitative data, we designed and synthesized 8 D–amino acid agonists for competitive testing in functional experiments. Dose-response titrations using MIP-1β production as a readout showed that gppqwnnpp (gpp) was the most potent activator of ALF3 (Figure 2A). The gppqwnnpp sequence incorporated the dominant residue in terms of signal strength at each sub-library position. It is also notable that gppqwnnpp bears no resemblance to GILGFVFTL in terms of primary sequence, barring the N-terminal glycine residue for which there is no chiral counterpart. Higher concentrations of gppqwnnpp peptide were required to induce activation of the ALF3 clone (Figure 2B) and target cell killing (Figure 2C) compared with the GILGFVFTL peptide. This reduced potency likely reflects decreased binding of D–amino acid peptides to MHC (see below). In further experiments, we demonstrated that the gppqwnnpp agonist could activate 4 clonotypically distinct GILGFVFTL-specific CD8^+^ T cell clones derived from genetically unrelated individuals (Figure 2D). Each of these T cell clones expressed TRBV19 with a unique TCRα chain and varying degrees of residue similarity in the third complementarity-determining region of the TCRβ chain (CDR3β) (Supplemental Figure 1B). These data demonstrate the power of combinatorial screening as a means to identify novel agonists.

### Protease and acid resistance of native versus synthetic agonists.

To elicit immune responses in vivo, antigenic structures must navigate host barriers associated with the route of entry, such as serum complement/proteases, gastric acid, and digestive enzymes. It is pertinent to note in this regard that strings of D–amino acids are thought to be sterically incompatible with protease-induced hydrolysis (13). We therefore compared the stability of gppqwnnpp and GILGFVFTL in human serum and simulated gastric acid as potential indicators of long-term biostability and immunogenicity. GILGFVFTL was rapidly degraded in human serum, reaching almost undetectable levels within 10 minutes (Figure 2E). In contrast, gppqwnnpp remained largely intact after 1 hour in human serum. Similar disparities were observed in simulated gastric acid (Figure 2F). These observations indicate that gppqwnnpp is likely to be highly stable in vivo, in contrast to GILGFVFTL.

### Functional and priming characteristics of native versus synthetic agonists.

**Figure 3:**
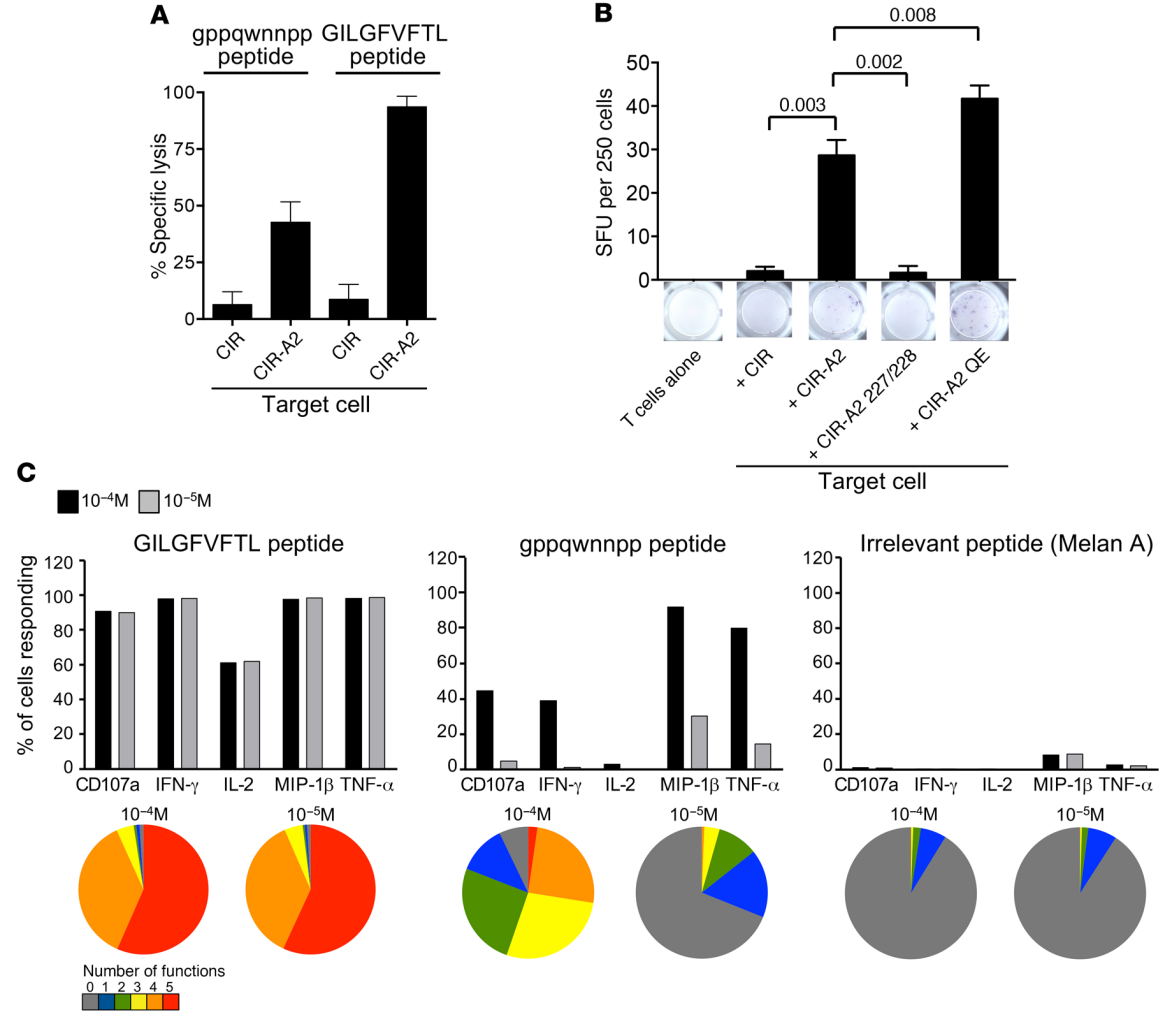
The synthetic agonist activates influenza virus matrix epitope-specific CD8^+^ T cells in the context of HLA-A2 and elicits polyfunctional outputs. (**A**) Chromium release cytotoxicity assay using the GILGFVFTL-specific CD8 T cell clone GD with CIR and CIR-A2 target cells. Effector/target cell ratio of 10:1. Peptide was added directly to the wells at 10^–5^ M and incubated for 5 hours. Errors from 3 replicates depict SEM. (**B**) ALF3 cells were seeded at 250 cells per well for an IFN-γ ELISpot plate. Each condition used 10^–4^ M gppqwnnpp peptide with ALF3 alone (250 cells per well) or with CIR-WT (A2^–^), CIR-A2, CIR-A2/D227K/T228A (227/228), or CIR-A2/Q115E (QE). The CIRs were used at 100,000 cells per well. Errors from 3 replicates depict SEM. Unpaired, 1-tailed *t* test with *P* values displayed. (**C**) Clonal GD CD8^+^ T cells were incubated with C1R-A2 with the indicated concentrations of GILGFVFTL (left), gppqwnnpp (middle), or ELAGIGILTV (right). Five distinct effector functions (CD107a, IFN-γ, IL-2, MIP-1β, and TNF-α) were measured using flow cytometry. Bars depict the percentage of CD8^+^ T cells expressing each function. Pie charts showing function are displayed below each corresponding bar graph. The pie segments represent the fraction of CD8^+^ T cells expressing the number of functions indicated in the key.

Next, we explored the lower functional sensitivity of gppqwnnpp relative to GILGFVFTL, hypothesizing that such differences may reflect a lack of traditional HLA-A2 anchor residues in the D–amino acid sequence, thereby destabilizing the binary pMHC-I complex. Using a T2 peptide binding assay (29), we observed no upregulation of HLA-A2 in the presence of gppqwnnpp (Supplemental Figure 1C), with no improvement following the addition of exogenous β2-microglobulin (data not shown) (30). Given the limited dynamic range of this assay (29), we sought to confirm epitope recognition using an endogenous epitope presentation system (Figure 3 and refs. 31–33). The GILGFVFTL-specific CD8 T cell clone GD killed gppqwnnpp-pulsed CIR cells transduced with HLA-A2 (C1R-A2), but did not kill WT C1R cells under the same conditions (Figure 3A). To minimize peptide cross-presentation among T cells, we next used enzyme-linked immunospot (ELISpot) assays with a limited number of T cells on a “carpet” of antigen-presenting cells, as used previously for examining the requirements for the presentation of pyrophosphate antigens to human Vγ9Vδ2 T cells (34). ELISpot with 250 ALF3 and 100,000 CIR-A2 cells per well revealed good responses to gppqwnnpp peptide (Figure 3B). Conversely, a carpet of WT C1R cells lacking HLA-A2 did not activate clonal ALF3 in parallel assays (Figure 3B). In addition, C1R-A2 targets with enhanced CD8 binding due to a Q115E mutation in the α2 domain of HLA-A2 (33) were effective presenting cells for gppqwnnpp, whereas C1R-A2 targets with abrogated CD8 binding due to a compound D227K/T228A mutation in the α3 domain of HLA-A2 (31) did not enable gppqwnnpp to activate ALF3. These observations show that gppqwnnpp is restricted by HLA-A2 and elicits functional outputs that are dependent on the interaction between HLA-A2 and the CD8 glycoprotein.

Next, we used intracellular cytokine staining to examine the agonist-induced functional profile of the CD8^+^ T cell clone GD in response to gppqwnnpp and GILGFVFTL. Five different effector outputs (CD107a, IFN-γ, IL-2, MIP-1β, and TNF-α) were measured simultaneously by flow cytometry in response to 2 different concentrations of gppqwnnpp, GILGFVFTL, and irrelevant Melan A peptide (sequence ELAGIGILTV) (Figure 3C). At 10^–4^ M, gppqwnnpp elicited multiple functions, with more than 80% of clonal GD cells expressing both MIP-1β and TNF-α. In line with the cytokine release and cytotoxicity data, however, a weaker profile was observed at 10^–5^ M. This loss of sensitivity likely relates to the weak affinity of gppqwnnpp for HLA-A2. In contrast, the native GILGFVFTL peptide elicited highly polyfunctional responses at 10^–4^ M and 10^–5^ M.

**Figure 4:**
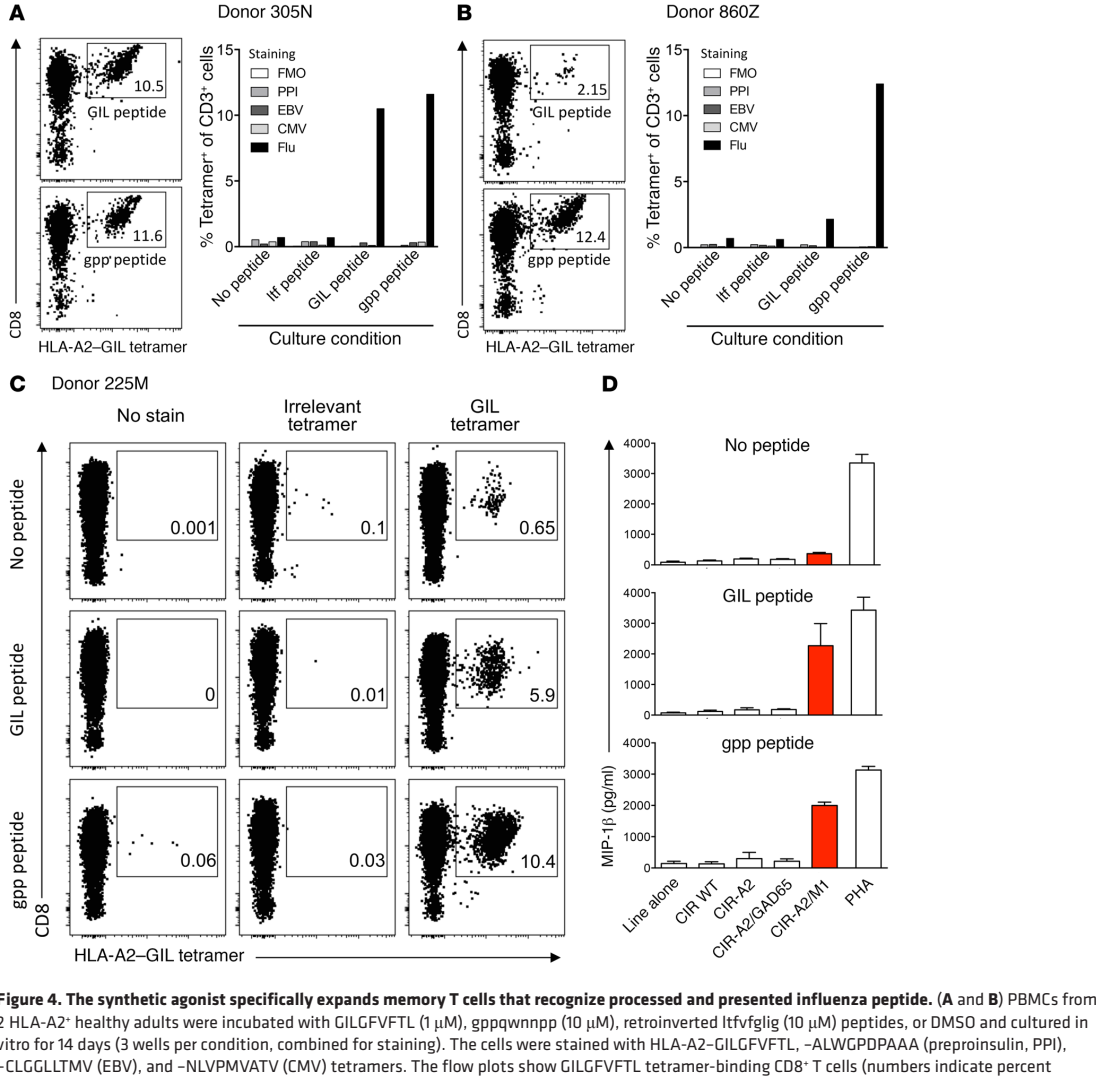
The synthetic agonist specifically expands memory T cells that recognize processed and presented influenza peptide. (**A** and **B**) PBMCs from 2 HLA-A2^+^ healthy adults were incubated with GILGFVFTL (1 μM), gppqwnnpp (10 μM), retroinverted ltfvfglig (10 μM) peptides, or DMSO and cultured in vitro for 14 days (3 wells per condition, combined for staining). The cells were stained with HLA-A2–GILGFVFTL, –ALWGPDPAAA (preproinsulin, PPI), –CLGGLLTMV (EBV), and –NLVPMVATV (CMV) tetramers. The flow plots show GILGFVFTL tetramer-binding CD8^+^ T cells (numbers indicate percent frequency within the total CD8^+^ T cell population). Data are summarized graphically for all other culture conditions and tetramer specificities. (**C** and **D**) A third set of PBMCs was primed with GILGFVFTL or gppqwnnpp and stained with irrelevant (ALWGPDPAAA, PPI) and GILGFVFTL tetramers. (**C**) Administration of DMSO alone, with no peptide, was also performed as a control. Each line was incubated overnight alone or with CIR-WT (A2-), CIR-A2, CIR-A2/GAD65, or CIR-A2/M1, or with PHA (in duplicate). (**D**) Supernatants were harvested and activation quantified by MIP-1β ELISA. Associated flow plots can be found in Supplemental Figures 2 and 3. Errors from 2 replicates depict SEM.

To extend these observations, we investigated the ability of gppqwnnpp to amplify GILGFVFTL-specific human memory T cells in vitro. Peripheral blood mononuclear cells (PBMCs) from healthy HLA-A2^+^ individuals were stimulated with either gppqwnnpp or GILGFVFTL for 14 days, and specific T cell expansions were quantified by flow cytometry after staining with a fluorochrome-labeled tetrameric HLA-A2–GILGFVFTL complex (Figure 4). Remarkably, we found that gppqwnnpp expanded comparable (donor 305N, Figure 4A) or even larger populations of tetramer-binding CD8^+^ T cells compared with GILGFVFTL (donor 860Z, Figure 4B and donor 225M, Figure 4C). Moreover, these effects occurred in the absence of bystander expansion (Figure 4, A and B and flow cytometry plots in Supplemental Figures 2 and 3). In addition, the T cell lines from donor 225M exhibited equivalent functional reactivity against C1R-A2 target cells expressing the full-length influenza A virus M1 protein, but did not respond to C1R-A2 cells expressing the irrelevant protein glutamic acid decarboxylase (GAD65) (Figure 4D). These data show that gppqwnnpp can expand GILGFVFTL-specific memory CD8^+^ T cells capable of recognizing the endogenously processed L–amino acid index peptide in the context of HLA-A2.

### T cell repertoire mobilization in response to native versus synthetic agonists.

**Figure 5:**
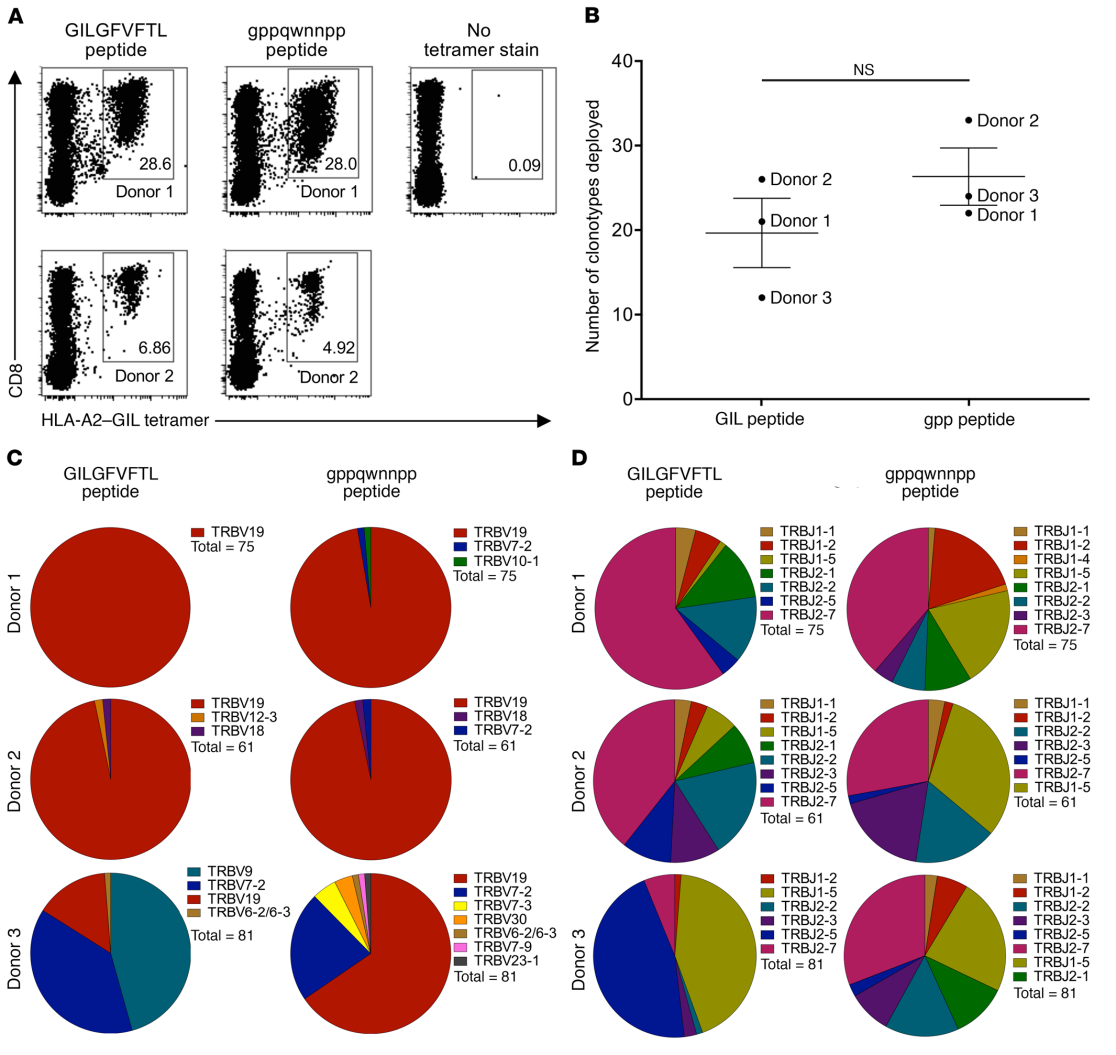
The synthetic agonist expands memory T cells expressing archetypal TCRs. (**A**) Healthy adult HLA-A2^+^ PBMCs were incubated with GILGFVFTL (10 μM) or gppqwnnpp (500 μM) and cultured in vitro for 14 days. HLA-A2–GILGFVFTL tetramer-binding cells were quantified by flow cytometry. Numbers denote the percent frequency of antigen-specific cells in the total CD8^+^ population. The stain control (no tetramer) is shown top right. (**B**–**D**) Viable HLA-A2–GILGFVFTL tetramer-positive CD3^+^CD8^+^ cells were sorted at greater than 98% purity from lines primed with GILGFVFTL or gppqwnnpp, and all expressed TRB gene rearrangements were characterized using a template switch–anchored RT-PCR with Sanger sequencing. The number of unique clonotypes (3 donors, **B**), TRBV gene usage (**C**), and TRBJ gene usage (**D**) are shown for each of 3 genetically unrelated donors. Random sampling was performed to normalize the data across different conditions.

A detailed understanding of the elicited TCR repertoire is an important consideration in the rational design of prototypic T cell vaccines (4). We therefore examined the clonotypic composition of antigen-specific memory CD8^+^ T cell populations expanded by gppqwnnpp and GILGFVFTL (Figure 5A). Three donors with a similar level of priming for the gppqwnnpp and GILGFVFTL peptides (Figure 5A for 2 of the donors) were used for clonotypic analysis of the T cell receptor repertoire. Using a fully quantitative template switch–anchored reverse transcription PCR (RT-PCR) in conjunction with Sanger sequencing (35), we found no significant difference in the number of clonotypes mobilized by these distinct peptides (Figure 5B). A strong bias toward the expansion of TRBV19^+^ clonotypes was observed in GILGFVFTL-stimulated cultures from donor 1 and donor 2 (Figure 5C), in line with previous studies of human naive and memory repertoires specific for this antigen (24, 27, 28). The corresponding gppqwnnpp-primed cultures displayed a similar gene bias, and overlapping TCRβ sequences across the 2 peptide conditions were detected within donors (Supplemental Table 1). Incongruously, the GILGFVFTL-stimulated culture from donor 3 was dominated by TRBV7-2^+^ and TRBV9^+^ clonotypes, which is unusual in the context of earlier work (24, 27, 28). In the same donor, however, gppqwnnpp remodeled these clonotypic expansions toward a more archetypal repertoire dominated by TRBV19^+^ sequences incorporating classical motifs in the CDR3β chain (Supplemental Table 1). Moreover, both gppqwnnpp and GILGFVFTL elicited public and near-public TRBV19^+^ clonotypes (Supplemental Table 1). We observed less-stringent TRBJ gene selection in these experiments (Figure 5D), again consistent with current knowledge (24, 27, 28). A bias toward TRBJ2-7^+^ clonotypes was nonetheless apparent in both agonist-primed cultures from donor 1 and donor 2, and a preference for TRBJ2-5 gene usage in the GILGFVFTL-primed culture from donor 3 was remodeled by gppqwnnpp toward a more conventional pattern, aligned with previous reports demonstrating frequent TRBV19/TRBJ2-7 gene rearrangements (24, 27, 28). The synthetic gppqwnnpp agonist therefore mobilizes antigen-specific CD8^+^ T cell repertoires that closely mimic those elicited by the native peptide.

### Structural conformation of native versus synthetic agonists.

**Figure 6:**
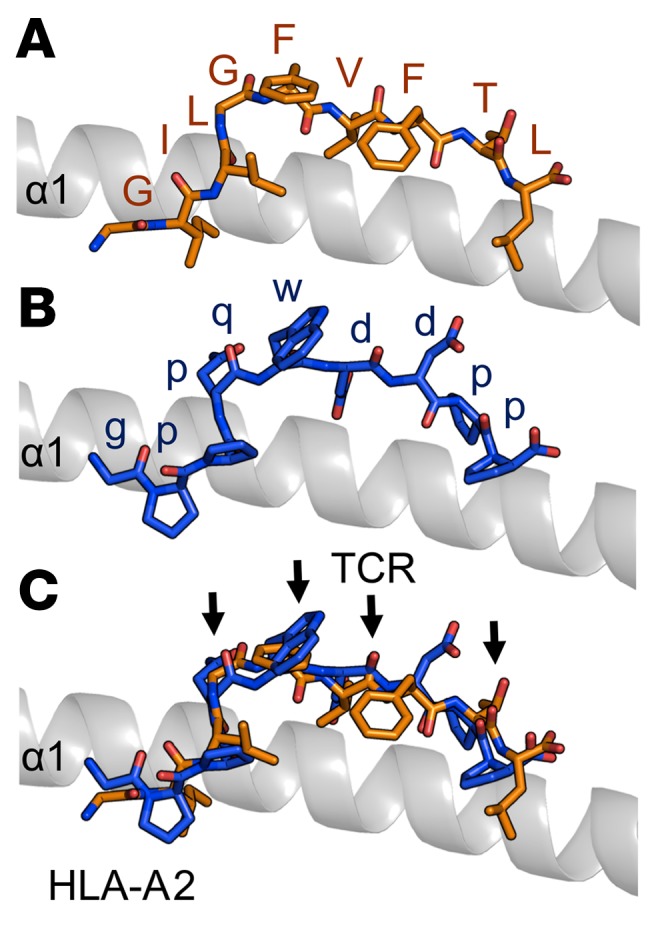
Structural modeling indicates that the native and synthetic agonists can form similar overall conformations. (**A**) Side view of the GILGFVFTL peptide (orange sticks) in the HLA-A2 binding cleft (gray cartoon). (**B**) Side view of the gppqwnnpp peptide (blue sticks) in the HLA-A2 binding cleft (gray cartoon). The structure was modeled in WinCoot using the JM22 TCR–HLA-A2–GILGFVFTL ternary structure as a reference. (**C**) Superposition of the GILGFVFTL peptide (orange sticks) and the gppqwnnpp peptide (blue sticks) in the HLA-A2 binding cleft (gray cartoon). Arrows demonstrate the main TCR contact points based on the JM22 TCR–HLA-A2–GILGFVFTL complex.

To determine the molecular basis of agonist cross-recognition in this setting, we attempted to solve the binary structure of the HLA-A2–gppqwnnpp complex. Although refolded protein yields were very low, presumably reflecting the weak affinity of gppqwnnpp for HLA-A2, we were able to generate small crystals. However, these crystals were not capable of diffracting to atomic resolution. We therefore modeled the HLA-A2–gppqwnnpp structure in silico (Figure 6, A and B), using the JM22–HLA-A2–GILGFVFTL ternary complex as a guide (36). The model indicated that gppqwnnpp could be presented by HLA-A2 in an overall conformation similar to that of GILGFVFTL. In particular, the D–amino acid residues Glu4, Trp5, Asp6, and Pro8 were solvent exposed, mimicking in 3 dimensions the main TCR contact residues identified in the JM22–HLA-A2–GILGFVFTL complex (Figure 6C and ref. 36). Thus, despite a lack of sequence homology between gppqwnnpp and GILGFVFTL, both antigens may look similar in terms of shape complementarity.

### The synthetic agonist effectively primes T cell responses that can protect from lethal influenza challenge.

**Figure 7:**
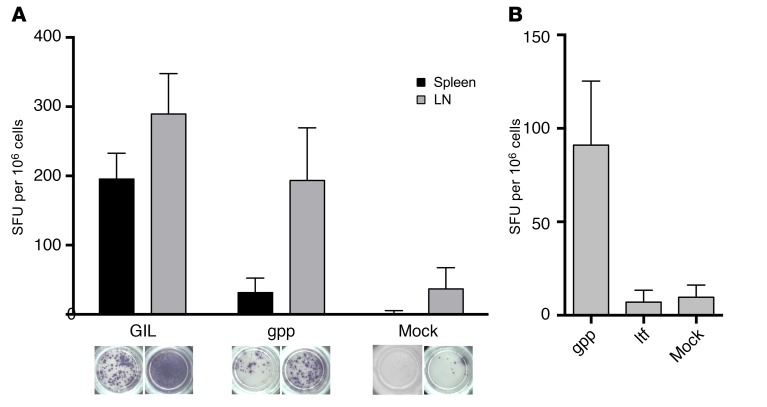
Vaccination of mice with the synthetic agonist elicits influenza-specific T cells. (**A**) HHD mice were primed (day 0) and boosted (day 14) via subcutaneous injection with 200 μl of a PBS preparation containing 100 μg GILGFVFTL (*n* = 4), gppqwnnpp (*n* = 4), or DMSO (Mock, *n* = 2) and 100 μl of incomplete Freund’s adjuvant. Single-cell suspensions were generated from spleens and peripheral lymph nodes (LNs) harvested on day 21, and GILGFVFTL-reactive cells were quantified in direct ex vivo IFN-γ ELISpot assays. Representative ELISpot wells are shown under each bar with triplicates performed per condition. Data are shown for a single experiment repeated with similar results (*n* = 3). Background values in the absence of peptide were subtracted. (**B**) Using the same approach as in **A**, with gppqwnnpp (*n* = 2), ltfvfglig (retroinverted GILGFVFTL peptide, *n* = 2), or DMSO (Mock, *n* = 2). Errors from 3 replicates depict SD.

To assess the biological relevance of these observations, we tested the ability of gppqwnnpp to prime effective de novo responses from the naive T cell pool. We used transgenic HLA-A2 mice (HHD mice) for this purpose, based on earlier work in similar transgenic murine systems (37). Mice were injected on day 0 and day 14 with GILGFVFTL, gppqwnnpp, or an irrelevant HLA-A2–restricted L–amino acid peptide (ELAGIGILTV) in incomplete Freund’s adjuvant (IFA). Preliminary dosing experiments showed that gppqwnnpp was safe and nontoxic (data not shown). One week after the second injection, cells were harvested from the spleen and peripheral lymph nodes (PLNs). Using direct ex vivo IFN-γ ELISpot analysis, we found that gppqwnnpp induced a GILGFVFTL-specific response in vivo, detectable most prominently in the PLNs (Figure 7A). No such response was observed with the ltfvfglig retroinversion of the GILGFVFTL L-peptide sequence (Figure 7B).

**Figure 8:**
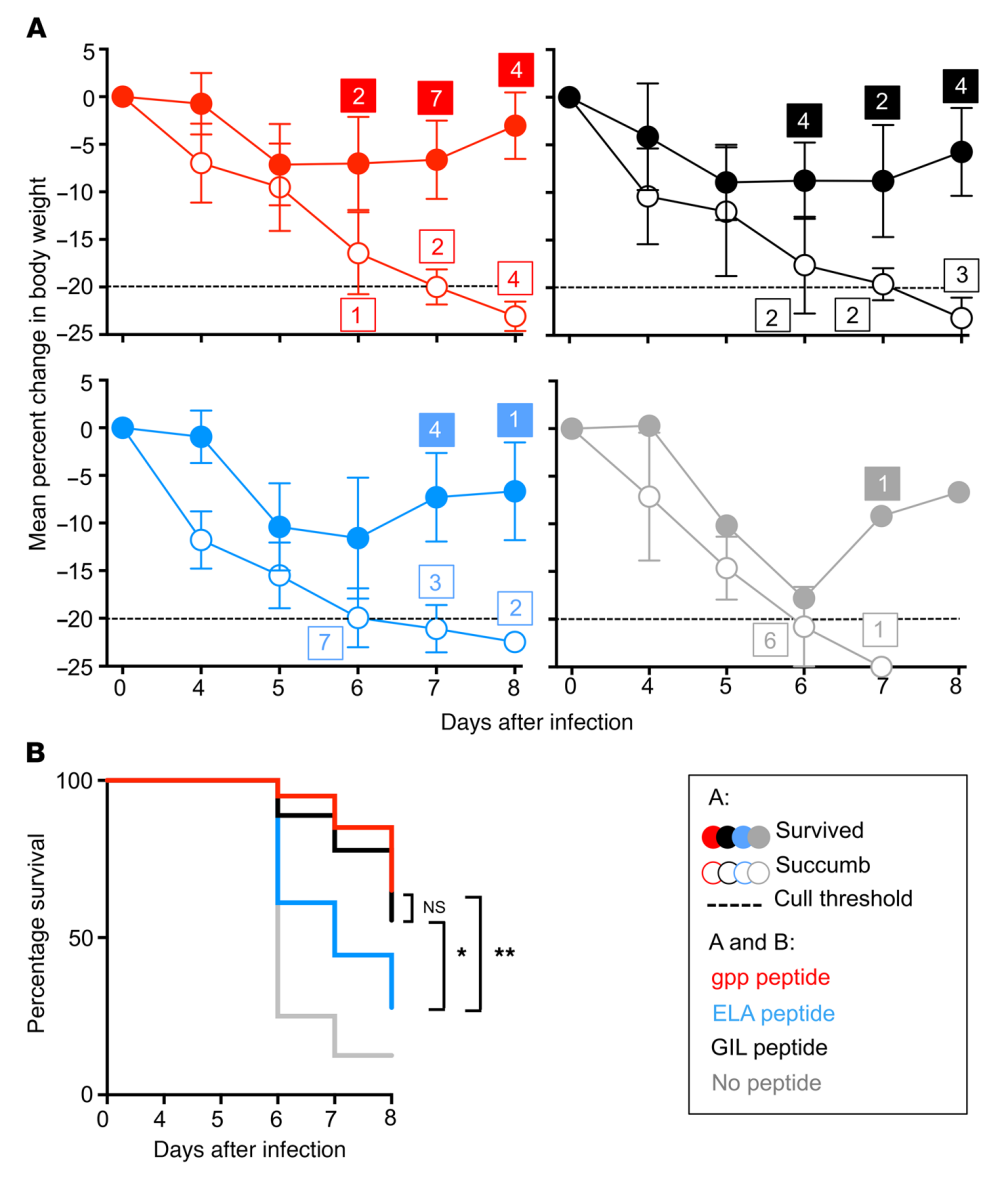
Vaccination with the synthetic agonist protects humanized mice against challenge with influenza A virus. (**A**) HHD mice were primed (day 0) and boosted (day 14) via subcutaneous injection with 200 μl of a 1:1 PBS and incomplete Freund’s adjuvant preparation containing 100 μg gppqwnnpp (red, *n* = 20), GILGFVFTL (black, *n* = 17), or ELAGIGILTV (blue, ELA, *n* = 17). A further group of mice remained unvaccinated (gray, *n* = 8). Mice were infected with PR8 on day 21 (females, 50 PFU; males, 100 PFU; Supplemental Figure 5). Body weight was recorded daily after infection and mice were classified as nonsurvivors and euthanized if their body weight fell by 20% or more (dotted line). By day 8, mice had either been euthanized (white circles) or gained weight and survived (red, black, gray, and blue circles). The number of mice euthanized on a given day is shown adjacent to the relevant data point. The number of mice that started to gain weight on a specific day is also shown. All mice that survived infection continued to gain weight for the duration of the assay and were euthanized on day 8. Error bars show SEM. (**B**) Survival graph for each group of mice based on the data in **A**. *P = 0.03; **P = 0.002 (unpaired 2-tailed *t* test). Data combined from 8 independent experiments.

To elaborate on these data, we vaccinated mice using the same regimen and performed intranasal challenge experiments with influenza A virus H1N1 strain A/Puerto Rico/8/34 (PR8). In accordance with local regulations, mice were euthanized if 20% or more of their initial body weight was lost, at which point the viral challenge was considered fatal. Female and male animals required 50 and 100 PFU, respectively, for 100% fatality (Supplemental Figure 4). On day 6 after PR8 infection, mice vaccinated with the control ELAGIGILTV peptide began to succumb rapidly (Figure 8A). In contrast, mice vaccinated with either GILGFVFTL or gppqwnnpp fared significantly better, with survival rates greater than 60% at day 8 (Figure 8B). It is also notable that we observed a trend toward better outcomes in the gppqwnnpp versus GILGFVFTL groups. This counterintuitive observation may reflect the greater in vivo stability and half-life of the D–amino acid peptide.

To extend these findings, we assessed the immunogenic effects of orally administered gppqwnnpp, which is stable in simulated gastric acid (Figure 2F). Mice received 3 doses of nonadjuvanted gppqwnnpp (300 μg total) in sodium bicarbonate at weekly intervals via oral gavage. One week after the final dose, cells were harvested from the mesenteric lymph nodes and tested for GILGFVFTL reactivity using IFN-γ ELISpot assays (Supplemental Figure 5A). Substantial GILGFVFTL-specific responses were detected in gppqwnnpp-vaccinated mice but not in mock-vaccinated controls. Further oral administration experiments using HHD mice showed that gppqwnnpp and GILGFVFTL were similarly immunogenic (Supplemental Figure 5B). Collectively, these experiments demonstrate that gppqwnnpp can prime protective immune responses in a humanized mouse model of influenza virus infection.

## Discussion

We used synthetic CPL arrays to design a nonnatural D–amino acid mimic of an immunodominant peptide epitope from the influenza virus matrix protein. This prototype agonist, gppqwnnpp, stimulated and expanded polyfunctional CD8^+^ T cells in vitro that cross-recognized the naturally presented L–amino acid epitope GILGFVFTL. Despite minimal sequence homology and nonclassical anchoring to HLA-A2, gppqwnnpp mobilized clonotypic repertoires in culture similar to those elicited by GILGFVFTL, in line with a structural model indicating common antigenic features and shape complementarity. Moreover, gppqwnnpp effectively primed GILGFVFTL-specific responses in naive, humanized mice, conferring protection from lethal influenza challenge. The stimulation of GILGFVFTL cross-reactive T cells by gppqwnnpp in 2 species that have very different TCR repertoires attests to how effectively HLA-A2–gppqwnnpp must mimic the key structural features of HLA-A2–GILGFVFTL. These findings validate an unbiased approach to the identification of synthetic ligands that could revolutionize the development of immunotherapies.

There is a clear strategic need to enhance the environmental and in vivo stability of T cell vaccines, both to minimize temperature-controlled supply chain burdens and to maximize biological efficacy. Previous attempts to optimize peptide-based interventions have been limited to the L–amino acid universe. For example, specific residues at MHC anchor or TCR contact sites can be replaced to enhance T cell activation and functionality (6, 26, 38, 39). Akin to their parent epitopes, however, such altered peptide ligands are rapidly destroyed in vivo by extracellular proteases and other components of various biofluids. In contrast, the D–amino acid peptide gppqwnnpp was vastly more stable than its natural counterpart in human serum and simulated gastric acid. Consistent with the latter finding, orally administered gppqwnnpp primed effector T cell responses in the gut-associated lymph nodes of humanized mice. It is notable in this regard that low-dose L–amino acid peptides delivered via the oral route may desensitize T cells, potentially enabling antigen-specific treatments for allergic and autoimmune conditions (reviewed in refs. 40, 41).

It is known that αβ TCRs can recognize many different peptides in the context of a single HLA molecule (18, 19, 21). This intrinsic degeneracy provides scope to construct novel ligands via the introduction of nonnatural side chains and/or inter-residue covalent bonds along the parent L–amino acid peptide backbone. Synthetic agonists offer potential advantages over subunit vaccines in terms of bioavailability, pharmacokinetics, and innate stability. Previous studies of L–amino acid peptides incorporating synthetic point mutations (D–amino acids, β–amino acids, nonproteolytic amino acids and pseudo-peptides, peptoids, and psi bonds) were designed to improve protease resistance and ligand binding to the presenting MHC molecule (17, 42–45). Other studies have used a retroinverso approach in which the L–amino acid peptide sequence is reversed using mirror image stereoisomer D–amino acids (46). In most cases, the modified peptides were more stable, both in the free state and bound to the relevant MHC molecule, and more immunogenic, both in vitro and in vivo (42–45, 47). D–amino acid agonists have also been explored in phase I/II clinical trials (48, 49). However, retroinverted D–amino acid peptides rarely mimic their parent antigens, and random synthetic component insertions can modify the immunogenicity profile of an L–amino acid blueprint (22).

In the present study, we overcome these limitations by using nonnatural CPL mixtures to screen rapidly and systematically for novel synthetic epitopes with defined agonist properties. Importantly, this platform is flexible and potentially applicable to any target antigen. Moreover, advances in solid-phase peptide synthesis (SSPS) (50) and the development of bioreactors that exploit organisms with expanded genetic codes (51) may enable industrial-scale production of synthetic T cell immunogens. Further effort is therefore warranted to translate the current proof-of-concept findings into real-world vaccine pipelines.

## Methods

### Human T cell clones and target cells.

The CD8^+^ T cell clones ALF3, GD, SG11, and SG25 were maintained in RPMI medium supplemented with 100 IU/ml penicillin, 100 μg/ml streptomycin, 2 mM l-glutamine, and 10% heat-inactivated FCS (R10), together with 25 ng/ml IL-15 (PeproTech) and 200 IU/ml IL-2 (Proleukin). All T cell clones were generated in-house. C1R–HLA-A*0201 (C1R-A2) cells were generated in-house as described previously (31) and maintained in R10. C1R-A2 cells were also lentivirally transduced to express the M1 protein from PR8 or human GAD65. The T lymphoblastoid hybrid cell line 0.174xCEM.T2 (T2), purchased from ATCC (CRL-19920), was maintained in R10.

### In vitro expansion of human T cells.

PBMCs were isolated by standard density gradient centrifugation from locally sourced venous blood samples or buffy packs obtained from the Welsh Blood Service (Pontyclun, United Kingdom). PBMCs were stimulated with various concentrations of peptide in R10. Progressively greater concentrations of IL-2 were added from day 2 to a maximum of 20 IU/ml by day 14. The cultures were then analyzed and sorted by flow cytometry.

### Combinatorial peptide library scans.

D–amino acid nonamer CPLs in positional scanning format (52) were manufactured at high purity using SPSS and HPLC (Pepscan Presto and GL Biochem). Prior to screening, CD8^+^ T cell clones were rested overnight in R2 (as for R10 but with 2% FCS). Target cells (6 × 10^4^ per well) were incubated in 96-well U-bottom plates with library mixtures (at 100 μM) in duplicate for 2 hours at 37°C. Clonal CD8^+^ T cells (3 × 10^4^ per well) were then added and the plates were incubated overnight at 37°C. Supernatants were harvested the following morning and assayed for MIP-1β by ELISA according to the manufacturer’s instructions (R&D Systems).

### Protease and acid stability.

Human serum from AB plasma (Sigma-Aldrich) was centrifuged for 10 minutes at 20,000 RCF to remove the lipid component. Serum supernatant was diluted to 25% in water (Merck Milli-Q system) and incubated for 15 minutes at 37°C. Triplicate samples of native and synthetic peptides (>93% pure; GL Biochem) were assayed simultaneously at a final concentration of 50 μg/ml after 1:20 dilution with 25% serum. Control reactions were set up as single tests with peptides diluted to the same concentration in Milli-Q water. Assays were run at 37°C. Samples of each peptide solution (100 μl) were removed at various time points and mixed with an equal volume of 15% aqueous trichloroacetic acid to precipitate serum proteins. Reactions were incubated for 40 minutes at 4°C and centrifuged for 5 minutes at 14,000 RCF. Supernatant was then stored at –20°C before analysis by liquid chromatography–mass spectrometry (LCMS). Three distinct ion fragments were monitored for each peptide. Stability was calculated as the area percentage of each serum-treated ion peak relative to the same ion peak at 0 minutes. Simulated gastric acid was prepared by dissolving 20 mg NaCl and 16 mg porcine pepsin (Sigma-Aldrich) in 70 μl concentrated HCl and diluting the solution to 10 ml with water (final pH 1.2). Mixtures were incubated for 15 minutes at 37°C. Triplicate samples of native and synthetic peptides were assayed as described above with dilution in simulated gastric acid. Control reactions were set up as single tests with peptides diluted to the same concentration in simulated gastric acid without pepsin. Assays were run at 37°C. Samples of each peptide solution (100 μl) were removed at various time points and stored at –20°C before analysis by LCMS.

### Cytotoxicity assays.

^51^Cr release assays were performed as described previously (53) using CIR or CIR-A2 cells as targets. Peptides were added directly to the wells and were present for the duration of the assay. Assays were run for 5 hours at 37°C.

### T2 peptide binding assay.

T2 cells lack the transporter associated with antigen processing (54) and require exogenous peptide to bind and stabilize MHC-I. Peptides (100 μM or 1 mM) were incubated with T2 cells (5 × 10^5^ per test) in R0 (as for R10 but lacking FCS) for 14 to 16 hours at 26°C. After an additional 2 hours at 37°C, the cells were stained for HLA-A2 surface expression with the monoclonal antibody BB7.2 (BD Biosciences). In some experiments, exogenous β2-microglobulin (AbD Serotec) was added during the incubation period (up to 150 μg/ml). Duplicate samples for each condition were acquired using a FACSCantoII flow cytometer (BD Biosciences). Data were analyzed with FlowJo software (Tree Star Inc.).

### pMHC-I tetramer staining.

Soluble biotinylated pMHC-I monomers were produced as described previously (55). Tetrameric pMHC-I reagents (tetramers) were constructed by the addition of phycoerythrin (PE)– or APC-conjugated streptavidin (Life Technologies, ThermoFisher Scientific) at a pMHC-I/streptavidin molar ratio of 4:1. CD8^+^ T cell clones or bulk cultures (5 × 10^4^) were incubated with PE- or APC-labeled tetramer (25 μg/ml) for 15 minutes at 37°C (reviewed in ref. 56) after staining with LIVE/DEAD Fixable Aqua (Life Technologies, ThermoFisher Scientific). Data were acquired using a FACSCantoII flow cytometer and analyzed with FlowJo software.

### Intracellular cytokine staining.

T cells were rested overnight at 1 × 10^6^ per ml in R2 (as for R10 with 2% FCS) and added to peptide-pulsed targets at an effector/target ratio of 1:2 in the presence of 5 μg/ml brefeldin A (Sigma-Aldrich), 0.35 μl/ml monensin (BD Biosciences), and 5 μl/ml αCD107a-FITC (clone H4A3, BD Biosciences). After 5 hours at 37°C, the cells were washed and stained with LIVE/DEAD Fixable Aqua followed by αCD3-PacificBlue (clone UCHT1, BioLegend), αCD8–APC-H7 (clone SK1, BD Biosciences), and αCD19-BV521 (clone HIB19, BioLegend). The cells were then fixed and permeabilized using a Cytofix/Cytoperm Kit (BD Biosciences) and stained intracellularly with αIFN-γ–PECy7 (clone 4S.B3), αTNF-α–PerCPCy5.5 (clone MAb11), αIL-2–APC (clone MQ1-17H12) (all from BioLegend), and αMIP-1β–PE (clone D21-1351, BD Biosciences). Data were acquired using a FACSCantoII flow cytometer and analyzed with FlowJo software. Cell population gates were set using fluorescence minus 1 staining controls as described previously (6).

### Clonotype analysis.

Viable tetramer-positive CD3^+^CD8^+^ cells were sorted at greater than 98% purity using a custom-modified FACSAria II flow cytometer (BD Biosciences). Molecular analysis of expressed TRB gene rearrangements was conducted using a template switch–anchored RT-PCR with Sanger sequencing technology as described previously (35).

### Structural modeling.

The structure of gppqwnnpp complexed with HLA-A2 was modeled in WinCoot (57) using the JM22 TCR–HLA-A2–GILGFVFTL ternary structure as a reference (36). The model was regularized using REFMAC5 (CCP4 Program Suite) (58). Figures were made using the PyMol Molecular Graphics System (Schrodinger LLC).

### Mice.

HHD mice were donated by Immunocore Ltd. or purchased from the Weatherall Institute of Molecular Medicine at the University of Oxford (Oxford, United Kingdom). These mice express a hybrid HLA-A2 transgene comprising the human α1/α2 domains and β2-microglobulin fused with a murine α3 domain (H-2D^b^) (59, 60). The HHD background strain was either C57BL/6J (subcutaneous experiments) or albino C57BL/6J Tyr^c–2J^ (oral experiments). Mice were housed throughout the study under specific pathogen–free conditions.

### Immunization, organ harvest, and influenza infection.

HHD mice were primed in the ventral inguinal area by injection with 200 μl of a PBS preparation containing 100 μg peptide (GILGFVFTL, gppqwnnpp, or ELAGIGILTV) and 100 μl IFA (Sigma-Aldrich). The same preparation was used to boost on the contralateral side 14 days later. Care was taken to ensure the formation of a raised area at the injection site, indicating that the vaccine was delivered subcutaneously rather than intraperitoneally. For experiments involving organ harvest, mice were euthanized 7 days after the last immunization, and the peripheral lymph nodes (inguinal, axial, brachial, and submandibular) were prepared as single-cell suspensions. For challenge experiments, mice were infected intranasally with influenza A virus strain PR8 obtained from the National Institute for Medical Research (London, United Kingdom). On the basis of dose optimization experiments, male mice received 100 PFU and female mice received 50 PFU PR8 in 50 μl sterile PBS under light anesthesia. Body weight was recorded daily after infection. Mice were classified as nonsurvivors and euthanized if their body weight fell by 20% or more. All other mice were euthanized 8 days after infection.

### Mouse and human IFN-γ ELISpot.

IFN-γ–producing cells were quantified using a mouse or human IFN-γ kit (MabTech). Briefly, ELISpot multiscreen filter plates (Millipore) were coated with capture antibody for 4 hours at 37°C, and then washed with PBS and blocked with R10 for 1 hour at room temperature. For mouse samples, 0.5 × 10^5^ or 2 × 10^5^ cells were added per well in the presence of peptide at a final concentration of 10^–5^ M. For human samples, 250 clonal T cells and 100,000 CIR WT or HLA-A2–transgenic cells were used per well. Medium alone was used as a negative control, and phytohemagglutinin (PHA) (1 μg/ml) was used as a positive control. Assays were incubated overnight at 37°C, and the plates were developed per the manufacturer’s instructions (MabTech). Spot-forming units (SFUs) were counted using an AID ELISpot Reader v5 (AID Diagnostika GmbH).

### Statistics.

For the human protease and simulated gastric acid assays, data in percentages were square-root transformed for all assays. Statistical analyses were conducted using unpaired *t* tests (1 per time point) corrected for multiple comparisons using the Holm-Sidak method (Alpha 0.05; Prism 6, GraphPad Software). For the human ELISpot assay, we used the unpaired, 1-tailed *t* test (Excel, Microsoft). For the HHD influenza survival curve, we used the unpaired, 2-tailed *t* test (Excel). *P* ≤ 0.05 was considered significant. Clonotype composition was compared using the Mann-Whitney *U* test (Prism 6, GraphPad Software).

### Study approval.

In vivo experiments were performed under United Kingdom Home Office approved projects (licenses 30/2355, 30/2635 and 30/3188) and conducted in compliance with the United Kingdom Home Office Guidance on the Operation of the Animals (Scientific Procedures) Act 1986. The use of human blood was approved by the School of Medicine Research Ethics Committee (Cardiff University School of Medicine, Cardiff, United Kingdom), project title: Comprehensive Analysis of T-cell Receptor Degeneracy and T-cell Crossreactivity (reference 12/09). Blood was sourced from local donors and the Welsh Blood Service (Pontyclun, United Kingdom). All human blood was procured and handled in accordance with the guidelines of Cardiff University’s Human Tissue Act compliance team, to conform to the United Kingdom Human Tissue Act 2004.

## Author contributions

JJM and AKS conceived and planned the project. JJM, MPT, GD, ESJE, SAEG, SG, BL, MC, JM, KL, KKM, TSW, KT, YW, HSL, RJC, JMP, MA, AL, AA, AG, PJR, JR, DKC, and DAP designed and performed experiments. JJM, MPT, SRB, DAP, and AKS drafted and critically revised the manuscript.

## Supplementary Material

Supplemental data

## Version 1 03/12/2018

Electronic publication

## Version 2 04/02/2018

Print issue publication
