# Supplementary material for: Peptide mimic for influenza vaccination using nonnatural combinatorial chemistry
Source: J Clin Invest. 2018 Mar 12;128(4):1569–80. doi: 10.1172/JCI91512 (PMC5873848; doi:10.1172/JCI91512)
Supplement: Supplemental data [file jci-128-91512-s001.pdf]

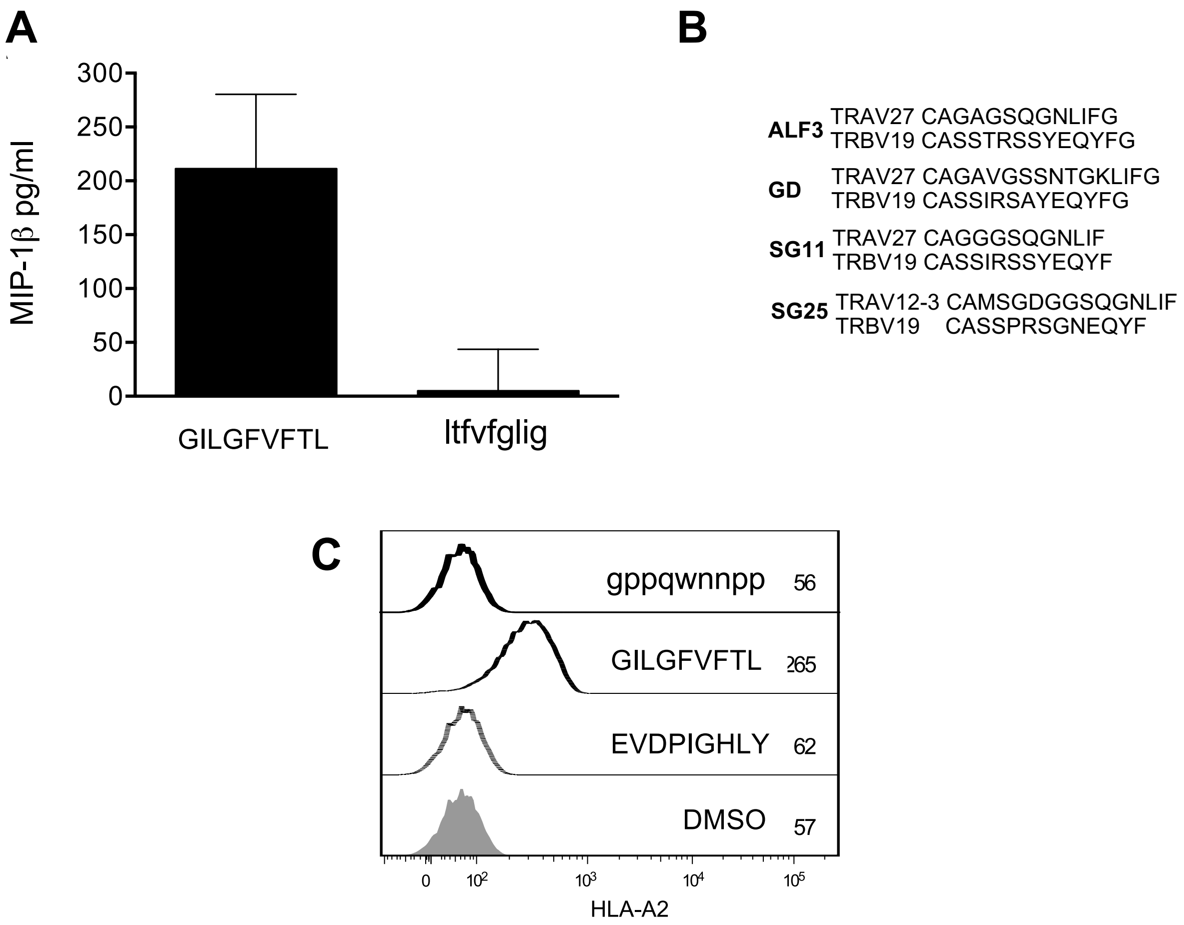

Supplementary Figure 1. (A) The GILGFVFTL retro-inverso D-peptide (sequence ltfvfglig) has no functionality. The CD8$^+$ T cell clone ALF3 was incubated with C1R-A2$^+$ targets with GILGFVFTL and ltfvfglig peptides (10^{-4}M) and MIP-1β release was quantified by ELISA after overnight incubation. **(B)** CDR3s of clone used in the study**.** TRAV and TRBV chain expression and CDR3s. **(C)** gppqwnnpp binding to HLA-A2 could not be detected at the surface of T2 cells. HLA-A2$^+$ T2 cells were incubated with 10^{-4} M GILGFVFTL, gppqwnnpp, EVDPIGHLY (HLA-A1 binding negative control) or DMSO and subsequently stained with anti-HLA-A2-FITC antibody to quantify binding strength of peptide to surface HLA-A2 molecules.

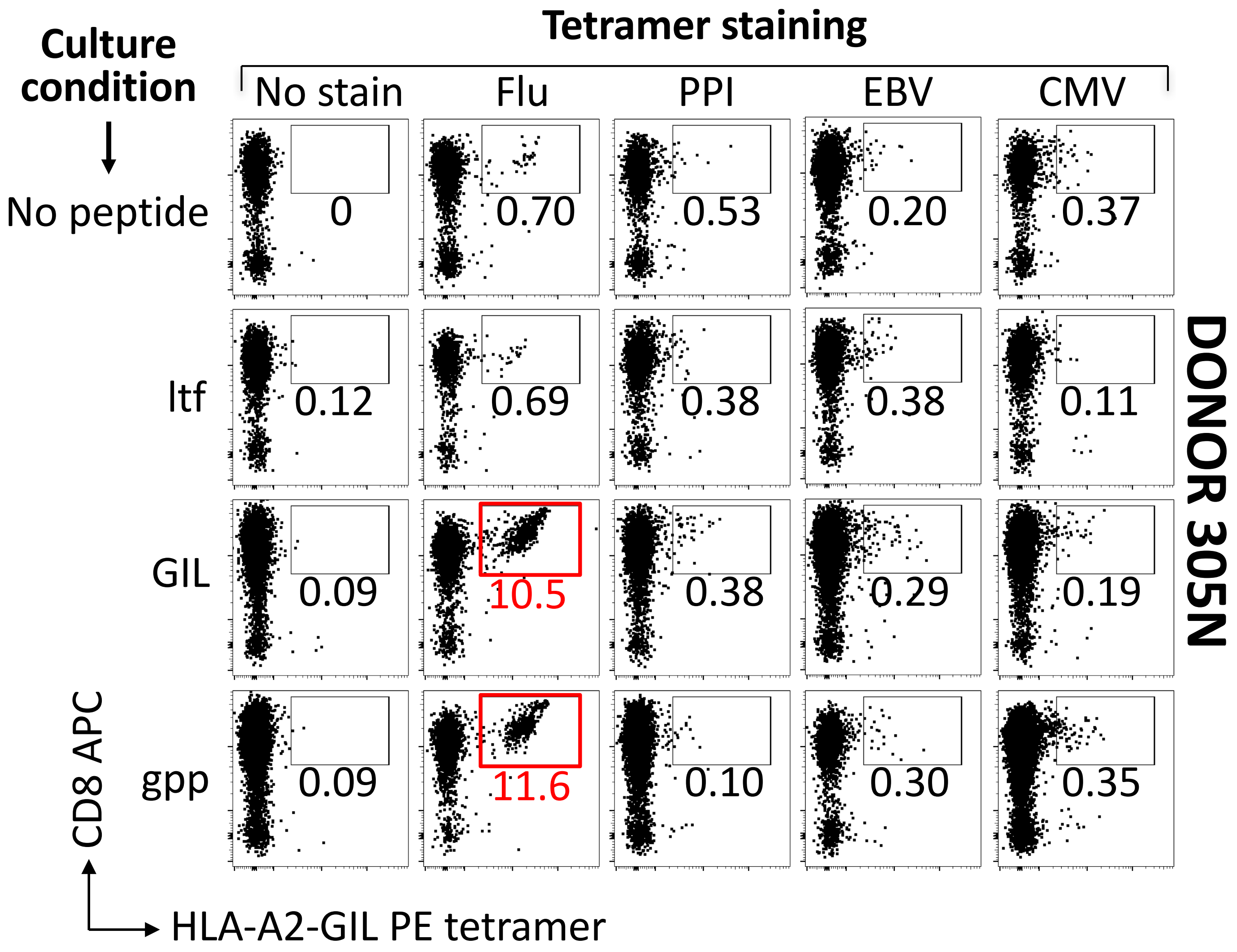

Supplementary Figure 2. The synthetic gppqwnnpp agonist specifically expands Influenza memory T cells. PBMCs from HLA-A2$^+$ healthy donor 305N were incubated with 1 µM GILGFVFTL (GIL), 10 µM gppqwnnpp (gpp), 10 µM retro-inverted ltfvfglig (ltf) peptides, or DMSO (no peptide) and cultured *in vitro* for 14 days. The cells were stained with HLA-A2-Flu (Influenza matrix, residues 58-66), -PPI (peproinsulin residues 15-24), -EBV (CLGGLLTMVL, MP2A residues 426-434) and -CMV (NLVPMVATV, pp65 residues 495-503) tetramers (**Figure 4A**). Cells were gated on lymphocytes and viable CD3+ cells. The percentage of cells residing in the tetramer+ gate is shown and if greater than 10% are displayed in red. The plots with red ink are also shown in Figure 4A.

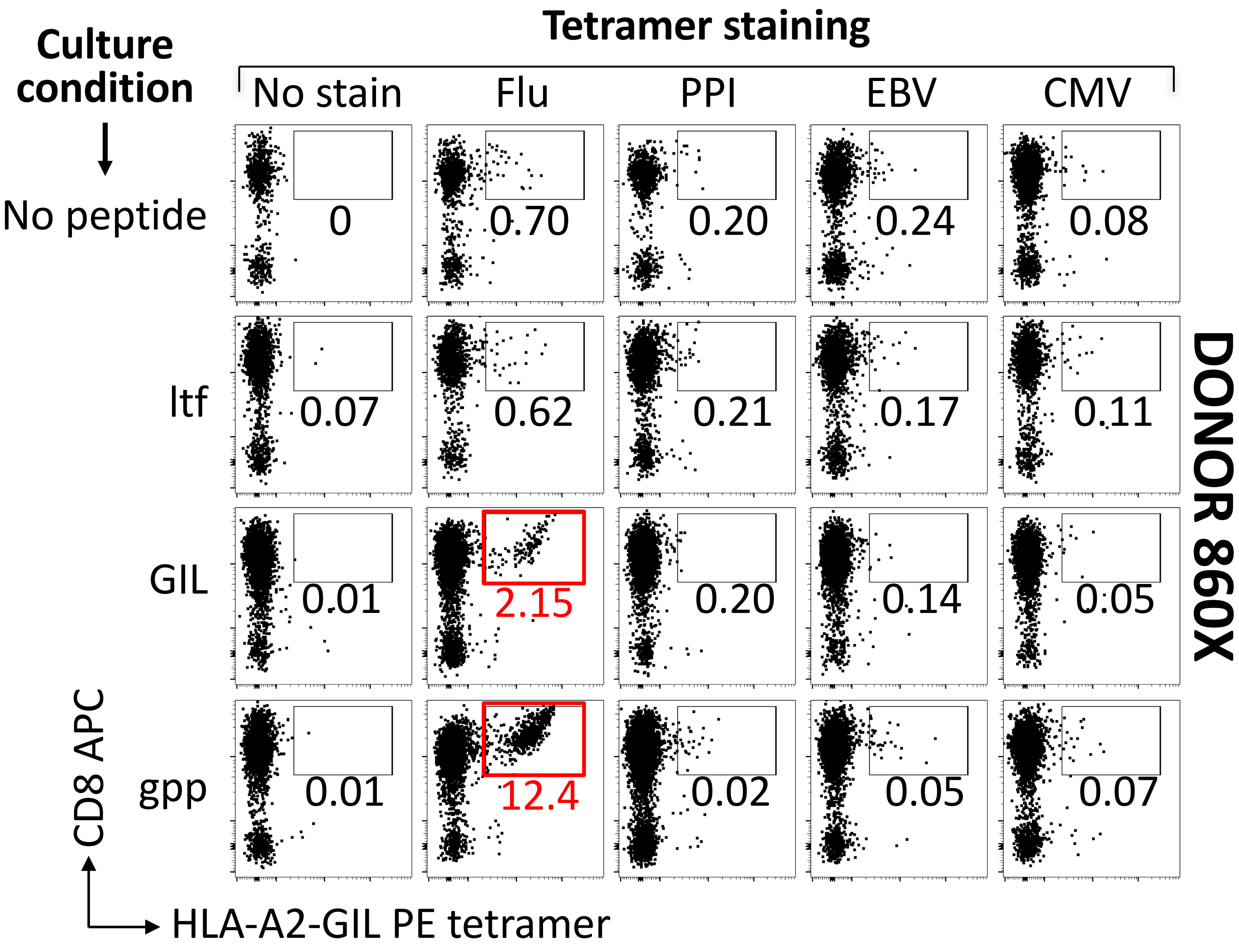

Supplementary Figure 3. The synthetic gpp agonist specifically expands Influenza memory T cells. PBMCs from HLA-A2+ healthy donor 860X were incubated with 1 µM GILGFVFTL (GIL), 10 µM gppqwnnpp (gpp), 10 µM retro-inverted ltfvfglig (ltf) peptides, or DMSO (no peptide) and cultured *in vitro* for 14 days. The cells were stained with HLA-A2-Flu (GILGFVFTL, Influenza matrix, residues 58-66), -PPI (peproinsulin residues 15-24), -EBV (CLGGLLTMVL, MP2A residues 426-434) and -CMV (NLVPMVATV, pp65 residues 495-503) tetramers (**Figure 4A**). Cells were gated on lymphocytes and viable CD3+ cells. The percentage of cells residing in the tetramer+ gate is shown and if greater than 2% are displayed in red. The plots with red ink are also shown in Figure 4B.

GIL Donor 1

TRBV	CDR3	TRBJ	Freq (%)
19	CASSIRSSYEQY	2-7	41.0
19	CASSLRSSYEQY	2-7	11.5
19	CASSIRSTGELF	2-2	5.1
19	CASSTGLYGYT	1-2	5.1
19	CASSARSTGELF	2-2	3.8
19	CASSTRSTGELF	2-2	3.8
19	CASSSRSGAEQF	2-1	3.8
19	CASSSRSANEQF	2-1	3.8
19	CASSIRSEYEQY	2-7	2.6
19	CASSRRSGETQY	2-5	2.6
19	CASSFRSSAEQF	2-1	2.6
19	CASSIFLGAEAF	1-1	2.6
19	CASSSRSSYEQY	2-7	1.3
19	CASSMRSAYEQY	2-7	1.3
19	CASSVRASNEQY	2-7	1.3
19	CASSVRSSYEQY	2-7	1.3
19	CASSIRAANEQY	2-7	1.3
19	CASSIRSGETQY	2-5	1.3
19	CASSIRSGNEQF	2-1	1.3
19	CASSISGANQPQH	1-5	1.3
19	CASSFRSSDVAF	1-1	1.3

gpp Donor 1

TRBV	CDR3	TRBJ	Freq (%)
19	CASSIRSSYEQY	2-7	14.7
19	CASSISGANQPQH	1-5	14.7
19	CASSIGSYGYT	1-2	12.0
19	CASSIRSAYEQY	2-7	9.3
19	CASSIGEHGYT	1-2	6.7
19	CASSIRSGNEQF	2-1	5.3
19	CASSTRSSYEQY	2-7	4.0
19	CASSIRSGFEQF	2-1	4.0
19	CASSTSGANQPQH	1-5	2.7
19	CASSNRAAYEQY	2-7	2.7
19	CASSRRSSYEQY	2-7	2.7
19	CASSSRSSYEQY	2-7	2.7
19	CASSDRAADTQY	2-3	2.7
19	CASSGRSVGELF	2-2	2.7
19	CASSARSTGELF	2-2	2.7
19	CASSILSNQPQH	1-5	2.7
7-2	CASSLVGLSYEQY	2-7	1.3
19	CASSVRSSYEQY	2-7	1.3
19	CASSIRSTDTQY	2-3	1.3
19	CASSTRSSGELF	2-2	1.3
19	CASSIRSNEKLF	1-4	1.3
10-1	CASRGIEAF	1-1	1.3

GIL Donor 2

TRBV	CDR3	TRBJ	Freq (%)
19	CASSIRSSYEQY	2-7	25.0
19	CASSIRSGETQY	2-5	8.3
19	CASSIRSEYEQY	2-7	6.9
19	CASSIRAGYEQY	2-7	5.6
19	CASSTRSTGELF	2-2	5.6
19	CASSSRSTGELF	2-2	5.6
19	CASSFRSSAEQF	2-1	5.6
19	CASSSHSTYEQY	2-7	4.2
19	CASSIRSTGELF	2-2	4.2
19	CASSIRSGNEQF	2-3	2.8
19	CASSSRASDTQY	2-3	2.8
19	CASSPYSADTQY	2-3	2.8
19	CASSANSNQPQH	1-5	2.8
12-3	CASSLFLAGDQPQH	1-5	1.4
19	CASSFRSGSNEQF	2-1	1.4
18	CASSETGTHTEAF	1-1	1.4
19	CASSTRSSYEQY	2-7	1.4
19	CASSMRSSHEQY	2-7	1.4
19	CASSSRSSYEQY	2-7	1.4
19	CASSTLGTDTQY	2-3	1.4
19	CASSARSTGELF	2-2	1.4
19	CASSIRSTDTQY	2-1	1.4
19	CAGSANSNQPQH	1-5	1.4
19	CASSFRSSDVAF	1-1	1.4
19	CASSTGLYGYT	1-2	1.4
19	CASSMGLYGYT	1-2	1.4

gpp Donor 2

TRBV	CDR3	TRBJ	Freq (%)
19	CASSGRSNQPQH	1-5	11.5
19	CASSIRSAYEQY	2-7	9.8
19	CASSISGANQPQH	1-5	8.2
19	CASSIRSSYEQY	2-7	8.2
19	CASSWLSNQPQH	1-5	6.6
19	CASSIRSTGELF	2-2	6.6
19	CASSPYSRANEQF	2-3	3.3
19	CASSDRAADTQY	2-3	3.3
19	CASSARSTGELF	2-2	3.3
19	CASSMGGGFAQPQH	1-5	1.6
19	CASSTTEVPNQPQH	1-5	1.6
19	CASSIRAGGNEQF	2-3	1.6
19	CASSSRASNEQY	2-7	1.6
19	CASSTRGAYEQY	2-7	1.6
19	CASSTRSSWEQY	2-7	1.6
19	CASSIRSNYEQY	2-7	1.6
19	CASSTRSSYEQY	2-7	1.6
19	CAGSGRAGGTQY	2-5	1.6
19	CASGIRSTDTQY	2-3	1.6
19	CASSTRSSDTQY	2-3	1.6
19	CASSPRSGDTQY	2-3	1.6
19	CASELTSTDTQY	2-3	1.6
19	CASSIRSGNEQF	2-3	1.6
19	CASSIRSTEVQF	2-3	1.6
19	CASSSRSVGELF	2-2	1.6
19	CASSDRSTGELF	2-2	1.6
19	CASSGRSVGELF	2-2	1.6
19	CASSLRATGELF	2-2	1.6
19	CASSILSNQPQH	1-5	1.6
19	CASSGRAGTEAF	1-1	1.6
18	CASSPVGVGEAF	1-1	1.6
19	CASSIGLYGYT	1-2	1.6
7-2	CAAVGEQY	2-7	1.6

GIL Donor 3

TRBV	CDR3	TRBJ	Freq (%)
9	CASSSDRDSTNSETQY	2-5	47.1
7-2	CASSFPGYQPQH	1-5	37.6
19	CASSTRSAYEQY	2-7	3.5
19	CASSIYSGNQPQH	1-5	2.4
6-2/6-3	CASSYNHRVTTSNQPQH	1-5	1.2
19	CASSIHQGAQGGYT	1-2	1.2
19	CASSIRSAYEQY	2-7	1.2
19	CASSTRTAYEQY	2-7	1.2
19	CASSLRSTDTQY	2-3	1.2
19	CASSIRSTDTQY	2-3	1.2
19	CASSIRSTGELF	2-2	1.2
19	CASSIYSNQPQH	1-5	1.2

gpp Donor 3

TRBV	CDR3	TRBJ	Freq (%)
7-2	CASSFPGYQPQH	1-5	22.2
19	CASSTRSAYEQY	2-7	13.6
19	CASSMRSAGELF	2-2	13.6
19	CASSIRSAYEQY	2-7	12.3
7-3	CASSPIGIDEQF	2-1	4.9
19	CASSIRSTDTQY	2-3	3.7
19	CASSTGAYGYT	1-2	3.7
30	CAWSLGGPDTQY	2-3	2.5
19	CASSIRSSYEQY	2-7	2.5
19	CASSLRSQETQY	2-5	2.5
19	CASSMRSSVEQF	2-1	2.5
23-1	CASSQWGDYPGPPSTDTQY	2-3	1.2
7-9	CASSLTTRGRTNSKNTEAF	1-1	1.2
19	CASTGGGGFGQPQH	1-5	1.2
30	CAWSEGDSTEAF	1-1	1.2
19	CASSVRSSYEQY	2-7	1.2
19	CASSGRSTDTQY	2-3	1.2
19	CASSMRSAGEMF	2-2	1.2
19	CASSIRSADGQF	2-1	1.2
19	CASSGRSAQEQF	2-1	1.2
19	CASSMRSSGEQF	2-1	1.2
19	CASSAGSYGYT	1-2	1.2
19	CASSTGACGYT	1-2	1.2
6-2/6-3	CTSSYSGGEQY	2-7	1.2

Supplementary Table 1: Viable HLA-A2-GILGFVFTL (GIL) tetramer+ CD3+ CD8+ cells were sorted at >98% purity from lines stimulated with GILGFVFTL or gppqwnnpp (gpp) peptide, and all *TRB* gene arrangements were characterized using a template-switch anchored RT-PCR with Sanger sequencing. TRBV and TRBJ gene usage, CDR3β amino acid sequence and clonotype frequency are shown for each of three genetically unrelated donors. Recurrent clonotypes are color coded.

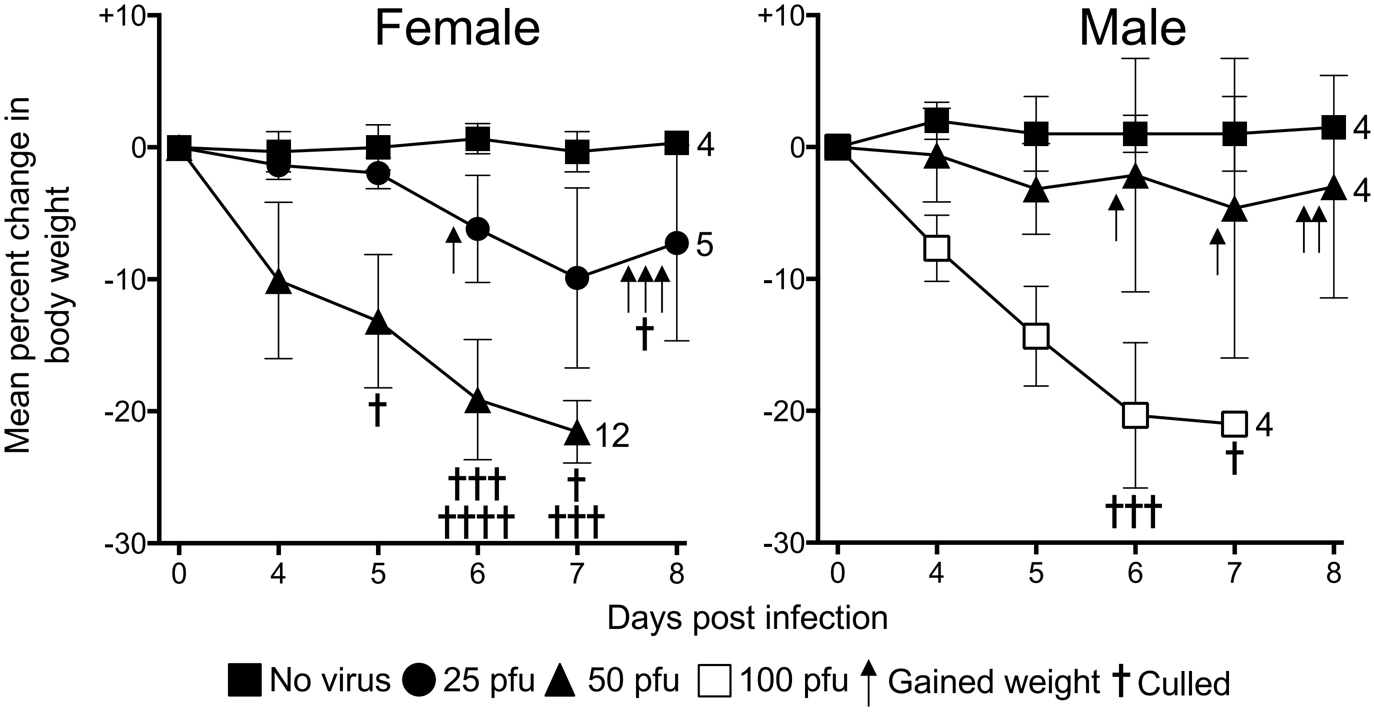

Supplementary figure 4: Establishing a lethal influenza viral dose for female and male C57BL/6 HHD mice. Groups of mice were left uninfected or infected with 25 or 50 plaque forming units (pfu) of PR8 influenza virus for female mice, and 50 or 100 pfu for male mice as indicated by the key. The number of mice per group is displayed at the end of the corresponding line on the graph. Mice were weighed before infection (day 0) and then on days 2, 4, 5, 6, 7 and 8 post infection with the data displayed for each time-point except day 2. According to Home Office UK enforcement mice were euthanized when they had lost 20%, or greater than 20% of their initial weight (day 0). A cross indicates the day on which an individual mouse was euthanized. For those mice infected with virus an arrow indicates the day that an individual mouse gained weight, and continued to gain weight for the duration of the experiment. All female mice receiving 50 pfu of virus had been euthanized by day 7, with all male mice receiving 100 pfu also being euthanized by day 7. As such, the aforementioned doses of virus for female and male mice, which gave 100% fatality, were used for the peptide vaccination component of this study (**Figure 8**).

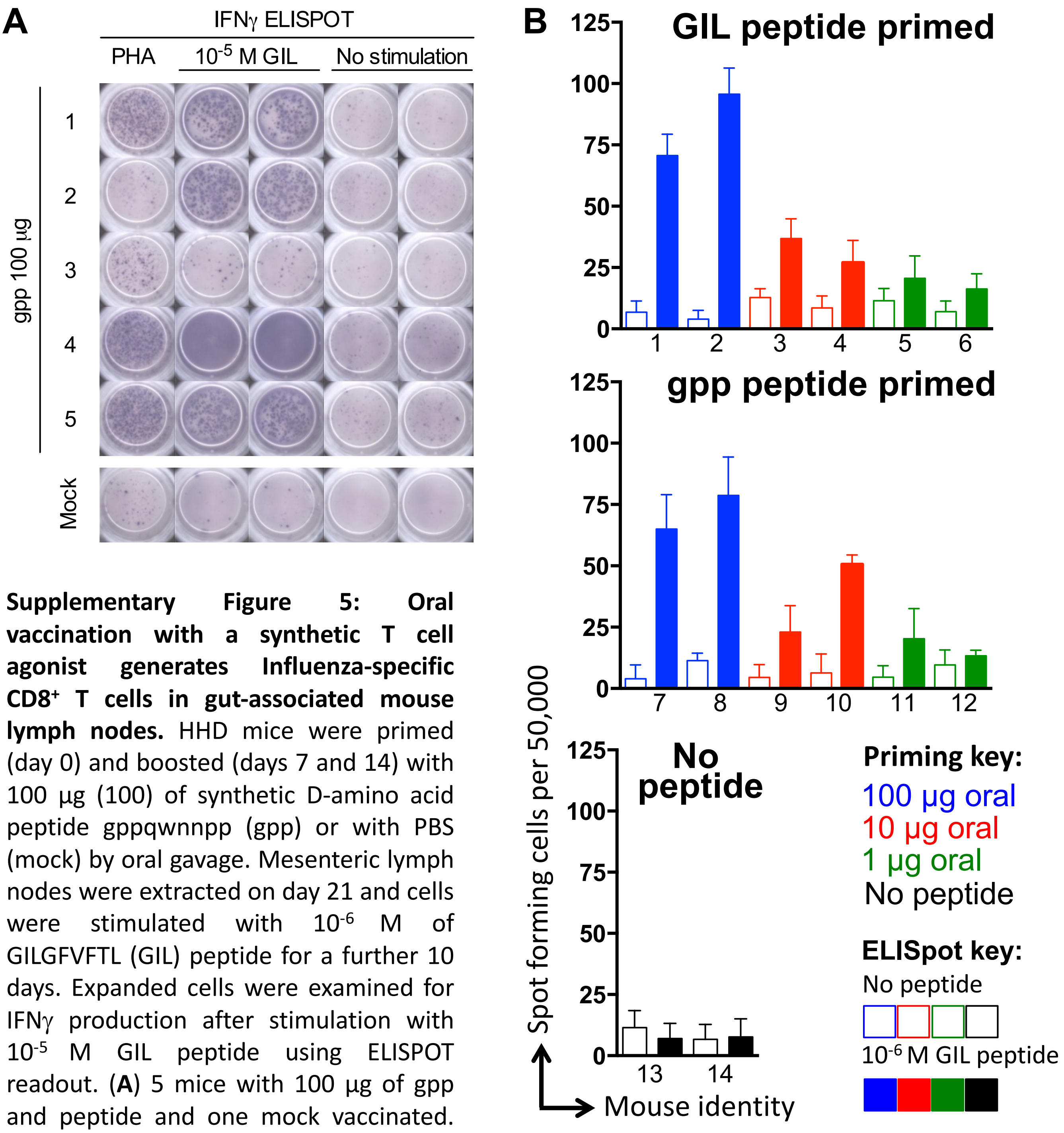

Supplementary Figure 5: Oral vaccination with a synthetic T cell agonist generates Influenza-specific CD8⁺ T cells in gut-associated mouse lymph nodes. HHD mice were primed (day 0) and boosted (days 7 and 14) with 100 µg (100) of synthetic D-amino acid peptide gppqwnnpp (gpp) or with PBS (mock) by oral gavage. Mesenteric lymph nodes were extracted on day 21 and cells were stimulated with 10^{-6} M of GILGFVFTL (GIL) peptide for a further 10 days. Expanded cells were examined for IFNγ production after stimulation with 10^{-5} M GIL peptide using ELISPOT readout. (**A**) 5 mice with 100 µg of gpp and peptide and one mock vaccinated. (**B**) 6 mice with GIL peptide at three concentrations (100, 10 and 1µg), 6 mice with gpp at three concentrations and 2 mock vaccinated mice. An equal mix of male and female mice were used for each condition. A minimum of three wells were used per condition.
